# Progress in Disease of Digestive Organs

**Published:** 1903-12-05

**Authors:** 


### Progress in Disease of Digestive Organs.

(*Continued from page* 153.)

Dysentery.—In South Africa the researches of Bruce [25] showed that the disease was probably bacillary, and not amœbic, but that no general infection of the organs occurred. No particular organism was obviously the causal agent, and there was not evidence enough to show that the normal intestinal flora became virulent. He showed that there was no relation between dysentery and enteric, the so-called post-enteric dysentery being probably a relapse of the original disease. In the recent epidemics in America the bacillus of Shiga is said to have been responsible for a mixed form of dysentery and diarrhœa, but Park,[32] who investigated the cases in New York State, found that the organism varied in different localities, both in cultural characteristics and in its reaction to serum. The agglutinative reaction was also uncertain, especially in children and in those suffering from other intestinal disease. Knox found that a large proportion of cases of summer diarrhœa of infants was due to Shiga's bacillus.[27] Amœbic dysentery has been studied by Futcher from records of 119 cases at Baltimore; [27] 9·4 of these occurred in children, and males were more liable than females. Hepatic abscess occurred in 20·3 per cent. of cases The disease has a great tendency to relapse, and the mortality is 24·5 per cent., whereas in 234 cases of the bacillary form recorded by Austin,[32] only 2 per cent. died. In this form, too, hepatic abscess is apt to occur late, as in a case in South Africa mentioned by Douglass, about a year after infection.[30] The treatment has been historically described by Lauder Brunton,[31] first by calomel, then later by castor oil and opium, then by ipecacuanha, and recently by sodium and magnesium sulphates, until thorough purging is produced. In South Africa, Douglass and others have used sulphur with remarkable success—half-drachm doses with Dover's powder every four hours in acute cases; Shipman and Smith,[32] Anders and Mason [27] advise calomel or castor oil followed by salines. All insist on the importance of irrigation of the bowel, but Anders believes it more efficacious in amœbic dysentery. He also speaks highly of ipecacuanha, as does Lauder Brunton for the Indian cases, but in South Africa Douglass found it useless. Calomel, opium, and ipecacuanha are not beneficial in children (Smith). All writers urge the value of warmth and rest in bed, and Anders considers that albumin water is a serviceable food. The local treatment consists in ointments, alternatively stimulant and sedative, to any ulcers which can be reached (Lauder Brunton), or in chronic cases curettage under an anæsthetic. Antiseptic douches are valueless (Shipman), but in amœbic dysentery quinine irrigations do some good (Futcher), while Burns [27] definitely ascribes some cases to the action of the malarial parasite. Gibson [32] has devised an operation by which an artificial valve is formed near the cæcum, and claims by this to be able to irrigate and medicate the colon more successfully. Manges [32] records four cases in which colotomy was adopted but only one had survived. He mentions a case of Boas in which a year was occupied in the cure, and one of Ewald's in which after six years another operation was necessary. He does not think Gibson's valve operation likely to be effective. Wiggin [32] has reported one amœbic case which was much benefited by operation. Though Shiga and Kinyoun have reported very favourably on the serum treatment, Park [32] has not found it very successful, perhaps owing to mixed infections.

Pancreatic Disease.—The relationship between disease of the pancreas and gall-stones has been shown by several observers. Opie [28] has pointed to the close anatomical relationship of the bile and pancreatic ducts as favouring the communication of disease. Weiner [29] quotes 32 cases, all fatal, in which the connection could be traced. Phillips [35] has recorded 12 cases of cancer of the pancreas associated with gall-stones, and mentions one of Perry and Shaw's. Stewart [33] believes that though pancreatitis usually is set up by bacterial infection plus obstruction of the duct by calculus the latter alone can produce a chronic interstitial inflammation. Flexner [28] considers that three-fifths of the cases of steatorrhœa are due to pancreatic disease, and notes that absence of bile in the intestine and excessive fat ingestion must be excluded. Phillips lays stress upon the fact that absence of ascites, coupled with jaundice and white stools, cannot be due to cancer of the liver and may be produced by either pancreatic disease or duodenal. In the latter case hæmorrhages occur early, whereas in pancreatic cancer they are late symptoms. Flexner considers laparotomy advisable in acute pancreatitis, and Radecki[28] considers that the bad results hitherto obtained were due to operation being resorted to too late. Weiner quotes a case in support of this view and urges operation before diagnosis. Radecki also favours operation in chronic cases, but Hale White[34] quotes figures to show that this is but seldom a fatal disease (four cases in 19,000 autopsies at Guy's), and mentions a case showing that spontaneous resolution may possibly occur. Glycosuria occurs in the interstitial form of pancreatitis (Opie, Flexner, Fitz), and its value in determining the cause of jaundice is considerable (Phillips).[35] The last named also gives reasons showing why the salol test (carboluria) is worthless as an indication of pancreatic disease. The occurrence of pancreatitis in mumps has been noted by Simonin and Cuche,[36] the symptoms being pain and tenderness in the epigastrium, with nausea, vomiting, fever, diarrhœa, and epistaxis in some cases. Occasionally in an epidemic the pancreas was the only gland affected. Tuberculosis of the pancreas and liver has been studied by Gilbert and Weil,[38] who found the former organ rarely affected, but differing from the liver in that a true cirrhosis was often found in chronic phthisis. Steinhaus[37] finds that chronic interstitial pancreatitis is frequent in connection with alcoholic cirrhosis of the liver, but not dependent on it; and Klippel and Lefas also consider that it is due to the direct action of the alcohol, although Hale White points out that it might be considered a result of backward pressure. Glycosuria only occurs when the pancreas is primarily attacked.

[25] Brit. Med. Jour., 1903, ii., p. 595. [27] Med. Rec., May 9, 1903. [28] Med. Rec., May 23, 1903. [29] Ibid., p. 817. [30] Dublin Jour. Med., April, 1903. [31] Lancet, 1903, ii, p. 7. [32] Med. Rec., 1903, ii., p. 75. [33] Amer. Jour. Med. Sci, May, 1903. [34] Brit. Med. Jour., 1903, ii., p. 126. [35] Lancet, 1903, i., p. 1796. [36] Lancet, 1903, ii., p. 772. [37] Pract., 1903, i., p. 372. [38] Ibid., p. 379.

*(To be continued.)*

